# Transcriptional mapping of the macaque retina and RPE-choroid reveals conserved inter-tissue transcription drivers and signaling pathways

**DOI:** 10.3389/fgene.2022.949449

**Published:** 2022-11-25

**Authors:** Ameera Mungale, David M. McGaughey, Congxiao Zhang, Sairah Yousaf, James Liu, Brian P. Brooks, Arvydas Maminishkis, Temesgen D. Fufa, Robert B. Hufnagel

**Affiliations:** ^1^ Medical Genetics and Ophthalmic Genomics Unit, National Eye Institute, National Institutes of Health, Bethesda, MD, United States; ^2^ Bioinformatics Group, National Eye Institute, National Institutes of Health, Bethesda, MD, United States; ^3^ Section on Epithelial and Retinal Physiology and Disease, National Eye Institute, National Institutes of Health, Bethesda, MD, United States; ^4^ Pediatric, Developmental and Genetic Ophthalmology, National Eye Institute, National Institutes of Health, Bethesda, MD, United States

**Keywords:** transcriptome, macula, fovea, retina, RPE

## Abstract

The macula and fovea comprise a highly sensitive visual detection tissue that is susceptible to common disease processes like age-related macular degeneration (AMD). Our understanding of the molecular determinants of high acuity vision remains unclear, as few model organisms possess a human-like fovea. We explore transcription factor networks and receptor-ligand interactions to elucidate tissue interactions in the macula and peripheral retina and concomitant changes in the underlying retinal pigment epithelium (RPE)/choroid. Poly-A selected, 100 bp paired-end RNA-sequencing (RNA-seq) was performed across the macular/foveal, perimacular, and temporal peripheral regions of the neural retina and RPE/choroid tissues of four adult Rhesus macaque eyes to characterize region- and tissue-specific gene expression. RNA-seq reads were mapped to both the macaque and human genomes for maximum alignment and analyzed for differential expression and Gene Ontology (GO) enrichment. Comparison of the neural retina and RPE/choroid tissues indicated distinct, contiguously changing gene expression profiles from fovea through perimacula to periphery. Top GO enrichment of differentially expressed genes in the RPE/choroid included cell junction organization and epithelial cell development. Expression of transcriptional regulators and various disease-associated genes show distinct location-specific preference and retina-RPE/choroid tissue-tissue interactions. Regional gene expression changes in the macaque retina and RPE/choroid is greater than that found in previously published transcriptome analysis of the human retina and RPE/choroid. Further, conservation of human macula-specific transcription factor profiles and gene expression in macaque tissues suggest a conservation of programs required for retina and RPE/choroid function and disease susceptibility.

## Introduction

The retina is a thin tissue of the posterior eye (range: 166.9 ± 20.9 μm–271.4 ± 19.6 μm (Grover et al., 2010) essential for processing light and transmitting information to the brain through the optic nerve. It is composed of multiple layers, which contain seven cell classes including rod and cone photoreceptors, in apposition with the retinal pigment epithelium (RPE), which is underlaid by the choroid. The choroid is a vascular structure that supports the outer retina, the RPE is necessary for maintaining healthy photoreceptors, and rods mediate dark-adapted vision while cones are responsible for color vision (Purves, 2001; Nickla and Wallman, 2010). The relative composition of cell types varies across the retina, and provides visual function specializations, such as high acuity color vision at the fovea, which is cone photoreceptor rich and rod depleted. Furthermore, the neural retinal layers change in thickness and morphology, including the inner and outer nuclear layers of the photoreceptors, whereas the choroidal layer has increased thickness at the macula but the morphology of the entire layer remains relatively unchanged (Bagci et al., 2008; Mori et al., 2016). Many genes have varied expression in different cell types and tissue layers, with 819 genes implicated in heritable retinal degenerations as of 2022 (RetNet, https://sph.uth.edu/RetNet/) (Daiger, 1998; Holt et al., 2015). While retinal degeneration may initiate in a single tissue layer, eventually the disease progresses across all tissue layers (Di Pierdomenico et al., 2017). Importantly, retinal manifestations in many diseases are region specific. For example, Stargardt disease and Best disease primarily affect the macula and cause loss of central vision first, whereas retinitis pigmentosa initiates in the rod-rich periphery (Walia and Fishman, 2009; MacDonald and Lee, 2013; Jones et al., 2018; Lamin et al., 2019). We hypothesize that this location-specific disease susceptibility of the retina is due to cellular specialization and region-dependent molecular interactions, which we can assess by regional layer-specific gene expression profiling of healthy retinae.

Previous investigations of the transcriptomic landscape of the human retina support a significant differential gene expression between the retina and the RPE/choroid/sclera tissue layers as well as across macular and peripheral regions, as described by Li et al. (2014). Comparison of gene expression between the nasal and temporal peripheries of retina and RPE/choroid/sclera tissues indicated little to no variability, yet macular and peripheral profiles remained distinct in each tissue (Li et al., 2014). Gene expression across the retina has shown to be consistent with the spatial distribution of photoreceptors and ganglion cells, and RPE-specific genes appear to be enriched in the periphery over the macula as outlined by Whitmore et al. (2014). Further characterization of gene expression between foveal and peripheral retina has been conducted using single-cell RNA sequencing in both primate (*Macaca fascicularis*) by Peng et al. (2019) and human retina by Voigt et al. (2019a). These studies showed variability in both cell type distribution and gene expression between foveal and peripheral retina (Voigt et al., 2019a; Peng et al., 2019). While it has been well established that regional differences exist across tissue layers in the primate retina, the molecular drivers of these differences remain unclear.

Here we generate an RNA-seq dataset from adult Rhesus macaque (*Macaca mulatta*) eyes that reflect the changing photoreceptor composition of the contiguous macular/foveal, perimacular, and peripheral regions to determine tissue- and location-dependent differential gene expression of the neural retina and RPE/choroid. The use of matched retina and RPE/choroid tissue biopsies in this analysis with an additional perimacular sample provides a contiguous assessment of gene expression across location as opposed to a binary macular vs. periphery comparison and allows for a more comprehensive look at patterns of gene expression and their related pathways and ontologies. We additionally explore conservation of these shared and distinct pathways through a meta-analysis comparing our macaque datasets to previously published human data. Finally, we examine gene regulation *via* transcription factors and tissue-tissue interactions using ligand/receptor analysis to understand differences in the macular and peripheral retina and the affiliated changes in the RPE/choroid.

## Materials and methods

### Animal care

All experimental protocols were approved by the National Eye Institute Animal Care and Use Committee. Procedures were performed in accordance with the United States Public Health Service policy on the humane care and use of laboratory animals.

### Tissue acquisition

Postmortem eyes were enucleated from two adult Rhesus monkeys (*M. mulatta*), aged 13 and 18 years. Two-millimeter punch biopsies were removed from macular/foveal, perimacular, temporal, and nasal regions of the retina. Following the biopsy, the tissues were separated into layers of neural retina and RPE/choroid using microdissection techniques. Biopsies were optimized for smallest diameter in other macaque samples in order to obtain the minimal number of cells required for RNA-sequencing. Optimization was performed by measuring total RNA yield in different sized biopsy samples as below and requiring a minimum of 200 ng as input for RNA-seq.

### RNA extraction

Total RNA was isolated from fresh tissues using the PicoPure™ RNA Isolation Kit (Applied Biosystems, Waltham, Massachusetts, United States) following mortar and pestle tissue homogenization. Extracted RNA samples were checked for quality using the 2100 Bioanalyzer (Agilent Technologies, Santa Clara, CA, United States) and only samples with RIN value > 6.5 were used (Range 1–10).

### RNA sequencing

Extracted RNA was prepared into 100 bp libraries for paired-end, poly-A-selected RNA sequencing on the Illumina HiSeq4000 at the NIH Intramural Sequencing Center. The raw sequence data is available at the Gene Expression Omnibus under accession GSE194285.

### Bioinformatic analysis

Briefly, reads were quantified against the macaque genome Mmul_8.0.1 using Salmon pseudo-alignment transcript quantification tool (version 0.13.0; Patro et al., 2017) (Patro et al., 2017), imported into R with tximport (Soneson et al., 2015) and analyzed for differential expression using DESeq2, using the Rhesus macaque sample as a covariate (Love et al., 2014). Transcript reads were additionally aligned to the human genome (GRCH38, gencode version 30 (Harrow et al., 2012), to supplement any unquantified genes. To select the gene name for each macaque transcript, we used the HGNC Comparison of Orthology Predictions (Yates, 2016) data, aggregated by the hcop R package (https://github.com/stephenturner/hcop). We counted the number of sources supporting each gene ID to transcript and selected the gene ID with the most support, as detailed in HYPERLINK "https://github.com/davemcg/macaque_macula_RNA-seq/blob/master/analysis/01_QC_prelim_analysis.Rmd" \o "https://github.com/davemcg/macaque_macula_RNA-seq/blob/master/analysis/01_QC_prelim_analysis.Rmd"https://github.com/davemcg/macaque_macula_RNA-seq/blob/master/analysis/01_QC_prelim_analysis.Rmdhttps://github.com/davemcg/macaque_macula_RNA-seq/blob/master/analysis/01_QC_prelim_analysis.Rmd.

Genes were considered to be differentially expressed based on IHW (Ignatiadis et al., 2016) corrected *p*-value < 0.05. Gene lists were then assigned gene ontologies using the clusterProfiler “enrichGO” function (Yu et al., 2012).

For the heatmap visualizations, we used length-scaled transcripts per million (TPM) quantification from tximport scaled by library size with the edgeR (Robinson et al., 2010) “calcNormFactors” function. The heatmaps were made with the R package ComplexHeatmap (Gu et al., 2016).

Human single cell transcriptome data from the scEiaD resource at plae.nei.nih.gov was searched for machine predicted RPE cells from fovea or peripheral punches. We found 217 cells, 159 from the fovea and 58 from the periphery, across four studies (Voigt et al., 2019b; Cowan et al., 2020; Voigt et al., 2020; Yan et al., 2020; Swamy et al., 2021). The Seurat object was downloaded on 14-01-2022 from plae.nei.nih.gov and subsetted to these 217 cells. The Seurat object was converted to a SingleCellExperiment object to run wilcox and t tests with the scran “findMarkers” test and used the study as a covariate. Genes were kept which had both a wilcox AUC (area under curve) greater than 0.7 (1 is best), an abs (log2) fold change greater than 0.7, and a FDR corrected *p*-value < 0.01. For the overlap testing we kept genes that had a padj < 0.01 in each comparison (single cell human RPE, bulk human RPE-choroid, or bulk macaque RPE-choroid) and the direction of the fold change between fovea and peripheral was the same. The venn diagram was created with the R package eulerr (https://cran.r-project.org/web/packages/eulerr/citation.html).

For the exact commands used in the analysis of this data, we make our Snakemake (Köster and Rahmann, 2012) reproducible workflow available at https://github.com/davemcg/macaque_macula_RNA-seq .

## Results

We performed RNA-seq on 2 mm retina and RPE/choroid tissues of 4 adult Rhesus macaque eyes in each of the central macular (foveal), perimacular, and peripheral regions (Supplementary Figure S1). Tissue dissections and RNA extractions were optimized for minimal input samples (<1 μg) to maximize spatial resolution. Principle component analysis (PCA) visualization indicates that samples group primarily by tissue. Foveal/macular and peripheral samples generally cluster furthest from each other, and perimacular samples cluster in between. Tissue from the nasal retina was also obtained and these samples cluster closest with perimacular samples. However, these were excluded from the analysis, as we were interested contiguous changes from the fovea/macula through the perimacula to the periphery (Supplementary Figure S2). We then assessed overall gene expression by tissue layer and location as well as differential gene expression between foveal/macular samples and other peripheral samples (Table 1).

**TABLE 1:** A summary of the differential gene expression (padj < 0.05) in retina and RPE/choroid by location.

Tissue	Location	# Genes
Retina	Fovea	6,921
	Perimacula	6,926
	Periphery	10,517
	Fovea vs. perimacula	4,633
	Fovea vs. periphery	9,644
RPE/choroid	Fovea	6,775
	Perimacula	7,465
	Periphery	10,983
	Fovea vs. perimacula	414
	Fovea vs. periphery	7,925

**FIGURE 1:**
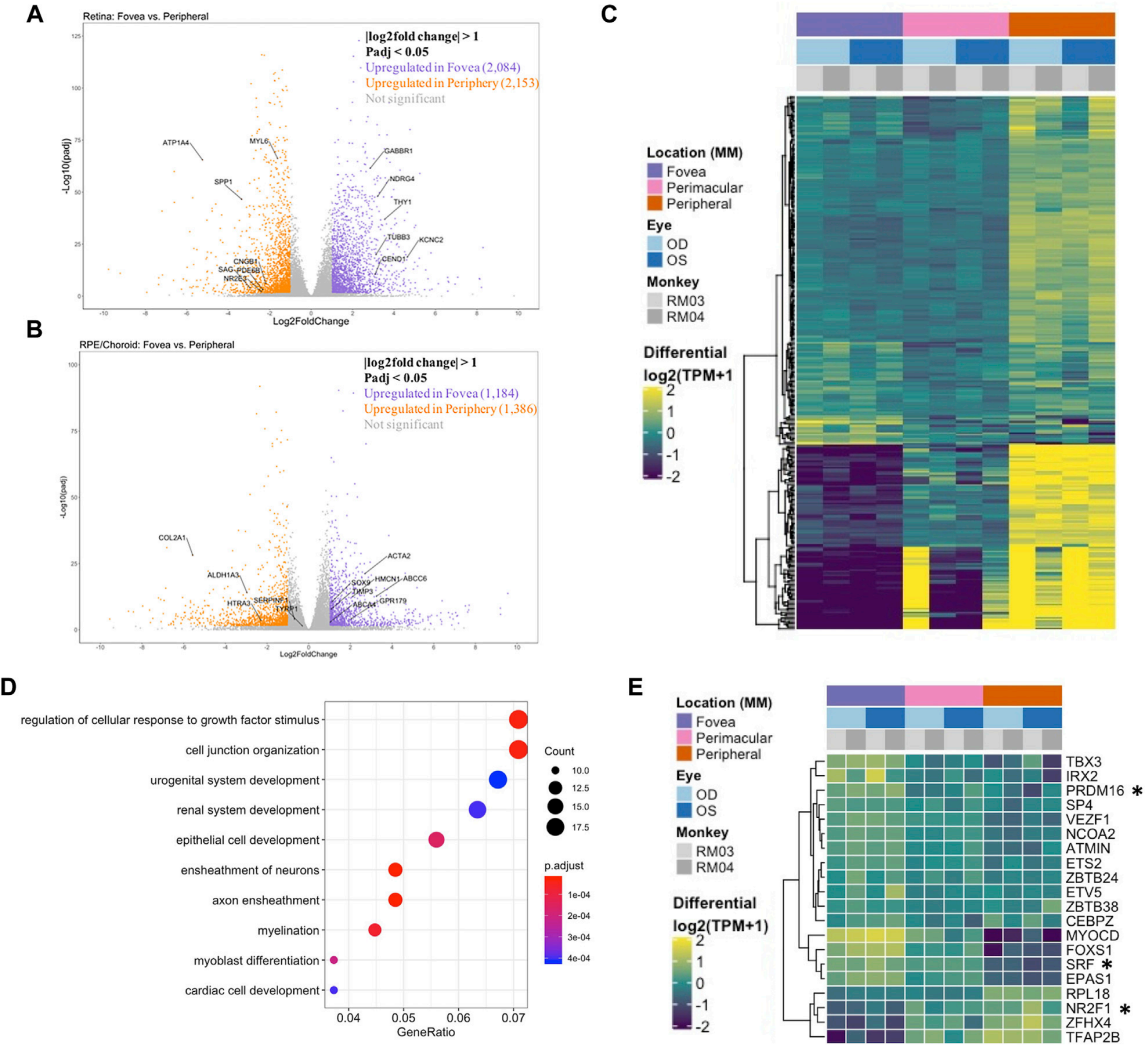
**(A)** A volcano plot depicting differentially expressed genes between foveal and peripheral tissues in the neural retina. Colored genes show significant up- or downregulation with several labeled genes having known tissue-specific roles in development and disease including PDE6B. **(B)** A volcano plot showing RPE fovea vs. periphery differential expression, with key genes labeled, including ABCA4 and TIMP3. **(C)** Unfiltered differential expression analysis of contiguously changing genes from fovea to perimacula to periphery in the RPE/choroid exhibits distinct blocks of lowly expressed genes in the fovea with a progressive increase in expression in peripheral tissues as well as blocks of genes with progressive downregulation moving outwards through the tissue despite less obvious morphologic changes in the tissue as compared to neural retina. **(D)** Top 10 enriched gene ontology (GO) terms from the RPE/choroid differentially expressed gene set include cell junction organization and epithelial cell development. VEGF is enriched in multiple of these ontologies and has increased expression in the macula over the periphery. **(E)** A subset of genes identified as differentially expressed transcription factors by location in the RPE/choroid. Changes in transcriptional regulators can be a factor in driving gene expression changes. *These transcription factors have published associations with the respective tissue in the literature.

Analysis of the foveal/macular versus peripheral regions in the retina and RPE/choroid depicted distinct sets of differentially expressed genes in each tissue layer, with 9644 and 7925 differentially expressed genes in the retina and RPE/choroid, respectively (Padj < 0.05) (Table 1). Many of the significantly differentially expressed genes are known to have tissue-specific roles in development and disease (Figures 1A,B). Retinal diseases also manifest uniquely at particular regions of the RPE or retina. For example, Age-Related Macular Degeneration (AMD) is thought to begin in the macular RPE (Boulton and Dayhaw-Barker, 2001), Late Onset Retinal Degeneration manifests in the peripheral RPE (Milam et al., 2000), Retinitis Pigmentosa causes photoreceptor loss in the peripheral retina (Jones et al., 2018), and finally, Cone-Rod Dystrophies primarily affect photoreceptors in the central retina (Hamel, 2007).

In order to confirm that the differentially expressed genes across locations further reflect cellular composition of different tissues, we used expression patterns of published rod-enriched genes as identified in Holt et al. (2015) and Mustafi et al. (2016). We observed correlated gene expression between eyes and animals, as well as a progressive increase in rod-enriched gene expression from the fovea/macula to the perimacula and then to the periphery, reflecting changes in neural retina composition (Supplementary Figure S3A). In this list of rod enriched genes, we find that our peripheral retina samples are 2.8 fold enriched in rod signal relative to the fovea/macula (*t*-test *p* < 3.5 e-12). The perimacular region was enriched in rod signal by 1.7 fold over the fovea/macula (*p* < 1.1 e-09). The enrichment of cone gene expression between the fovea/macula and peripheral retina samples was 1.6 fold (*p* < 0.02) (Supplementary Figure S3B; Supplementary Table S1). Among the RPE samples the enrichment of rod and cone markers was less substantial and not significant (Supplementary Figure S3C).

Next, we performed an unfiltered analysis of the differentially expressed, contiguously changing genes from fovea/macula to perimacula to periphery in the RPE/choroid. This analysis highlights stepwise changes due to changing photoreceptor composition in the cone-predominant macula, rod-predominant periphery, and admixed perimacula, and how these morphological changes in the neural retina may affect changing gene expression in the RPE/choroid. Differential expression analysis revealed distinct blocks of genes with low expression in the fovea/macula that increase moving outward through the perimacula to the periphery, and other genes that are highly expressed in the fovea/macula but progressively decrease moving outwards (Figure 1C). We observe enrichment for gene ontologies including cell junction organization and epithelial cell development, as well as urogenital and renal system development (Figure 1D).

Next, we sought to understand the transcriptional regulators driving the spatial and inter-tissue differences in gene expression. Using a list of all known transcription factors in humans, we identified a subset which is differentially expressed by location in each of the retina (Supplementary Figure S4B) and RPE/choroid datasets (Figure 1E). After conducting a literature review, we further identified transcription factors with known associations in the respective tissues. In the retina and RPE, distinct sets of fovea/macula -enriched and periphery-enriched transcription were elucidated. Our data shows three transcription factors, *NFRF1*, *IRX2*, and *ZFHX4*, to be differentially expressed in both neural retina and RPE/choroid and show a similar pattern in independent human data curated from eyeIntegration (Swamy and McGaughey, 2019) (Supplementary Figure S5). Interestingly, *ZFHX4* appears to be enriched in the macular neural retina and the peripheral RPE/choroid, whereas *NFRF1* and *IRX2* expression is present in both tissues.

**FIGURE 2:**
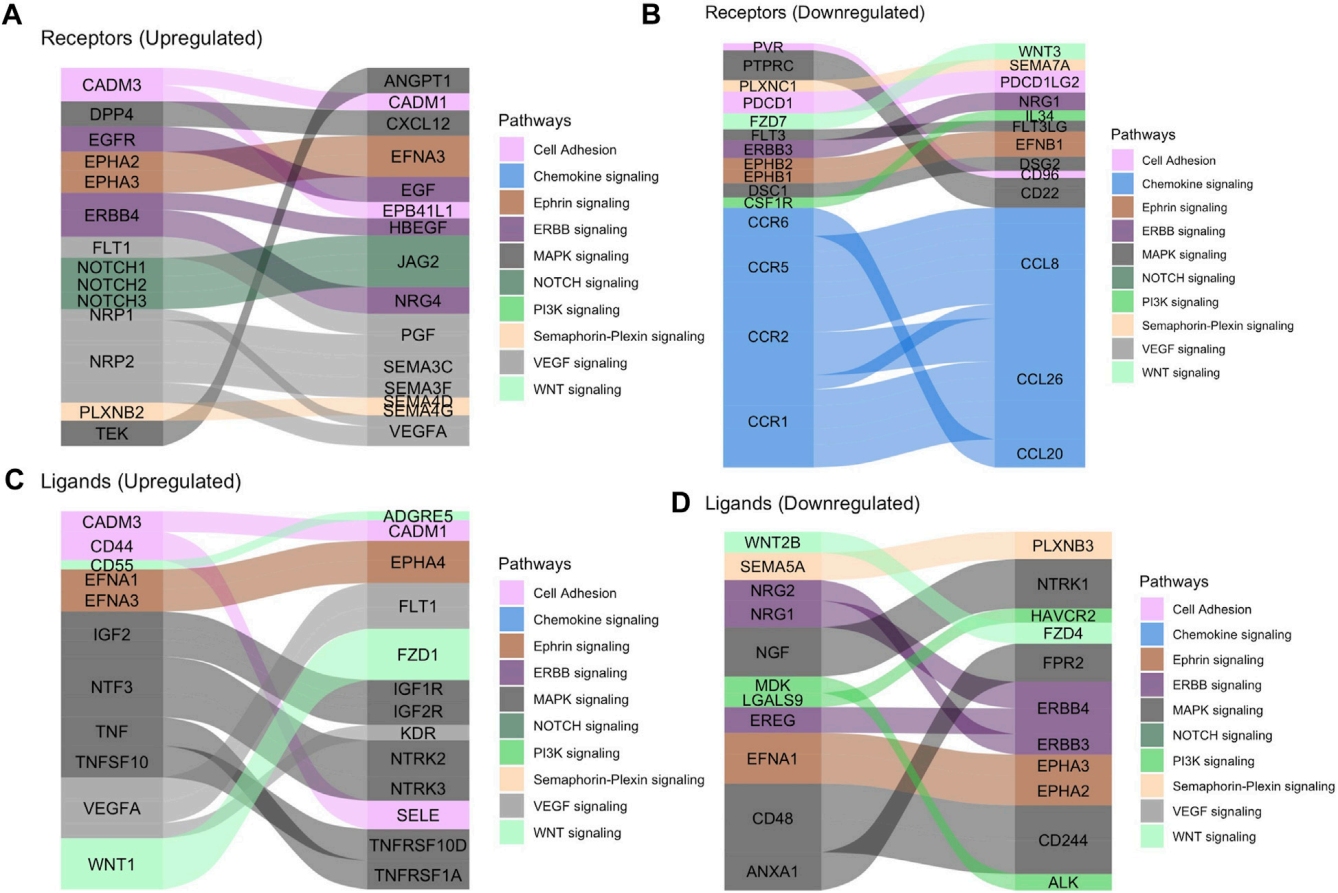
Tissue-tissue interactions showing receptor/ligand pairs in the central retina. **(A)** Upregulated macular receptors in the RPE/choroid are linked to their upregulated ligand in the macular neural retina. Interactors are grouped by signaling pathway and the width is scaled by average log2 fold change. **(B)** Downregulated macular receptors in the RPE/choroid and their respective downregulated ligand in the macular neural retina. **(C)** Upregulated macular ligands in the RPE/choroid are linked to their upregulated receptor in the macular neural retina. **(D)** Downregulated macular ligands in the RPE/choroid and their corresponding downregulated receptors in the macular neural retina.

We then analyzed tissue-tissue interactions using ligand and receptor characterizations from the CellPhoneDB database (Efremova et al., 2020). After converting protein names from the database to gene names *via* the Uniprot conversion tool and merging the data with our location-based differential expression, we generated lists of distinct up- and down-regulated interactions in which both interacting partners in each tissue were either up- or downregulated in the fovea/macula over the periphery (Table 2). Pathway analysis on these gene lists shows enrichment in the upregulated interactors for cell adhesion pathways (Figures 2A,C) and that downregulated interactors were highly enriched for Wnt signaling in kidney disease and the ErbB signaling pathway, both of which are heavily involved in tissue development, including ocular development (Figures 2B,D; Supplementary Figures S7C,D). Additionally, receptors involved in chemokine signaling were downregulated in the fovea/macula (Figure 2B).

**TABLE 2:** Ligand-receptor interactions across neural retina and RPE/choroid in the macula.

Upregulated interactions in the macula		Downregulated interactions in the macula	
RPE/choroid	Neural retina	RPE/choroid	Neural retina
NOTCH2	JAG2	EPHB2	EFNB1
FGFR1	NCAM1	WNT2B	FZD4
NTF3	NTRK3	EFNA1	EPHA2
IGF2	IGF1R	EPHB1	EFNB1
EPHA2	EFNA3	CCR1	CCL26
NOTCH1	JAG2	NRG2	ERBB3
PLXNB2	SEMA4D	CD48	CD244
NTF3	NTRK2	FLT3	FLT3LG
TNFSF10	TNFRSF10D	DSC1	DSG2
EPHA3	EFNA3	EFNA1	EPHA3
TEK	ANGPT1	PTPRC	CD22
VEGFA	FLT1	CCR5	CCL8
PLXNB2	SEMA4G	LGALS9	HAVCR2
NOTCH3	JAG2	MDK	ALK
WNT1	FZD1	PLXNC1	SEMA7A
NRP1	VEGFA	ANXA1	FPR2
EGFR	EGF	FGFR1	NCAM1
EPHA4	EFNB1	CCR1	CCL26
NRP2	SEMA3C	NGF	NTRK1
ERBB4	HBEGF	CD48	CD244
IGF2	IGF2R	FZD7	WNT3
EFNA1	EPHA2	CCR6	CCL20
EPHB1	EFNB1	CCR2	CCL26
TNF	TNFRSF1A	CCR2	CCL8
IGF2	IGF2R	PLXNB2	SEMA4C
EFNA3	EPHA4	PVR	CD96
CD55	ADGRE5	NRG1	ERBB4
CD44	SELE	ERBB3	NRG1
ERBB4	NRG4	PTPRC	CD22
CADM3	CADM1	SEMA5A	PLXNB3
EFNA1	EPHA3	CSF1R	IL34
CADM3	EPB41L1	CCR5	CCL8
VEGFA	FLT1	PDCD1	PDCD1LG2
VEGFA	KDR	CCR1	CCL8
NRP2	PGF	EREG	ERBB4
DPP4	CXCL12		
EPHB6	EFNB1		
NRP2	VEGFA		
FLT1	PGF		
EFNA1	EPHA4		
NRP2	SEMA3F		

**FIGURE 3:**
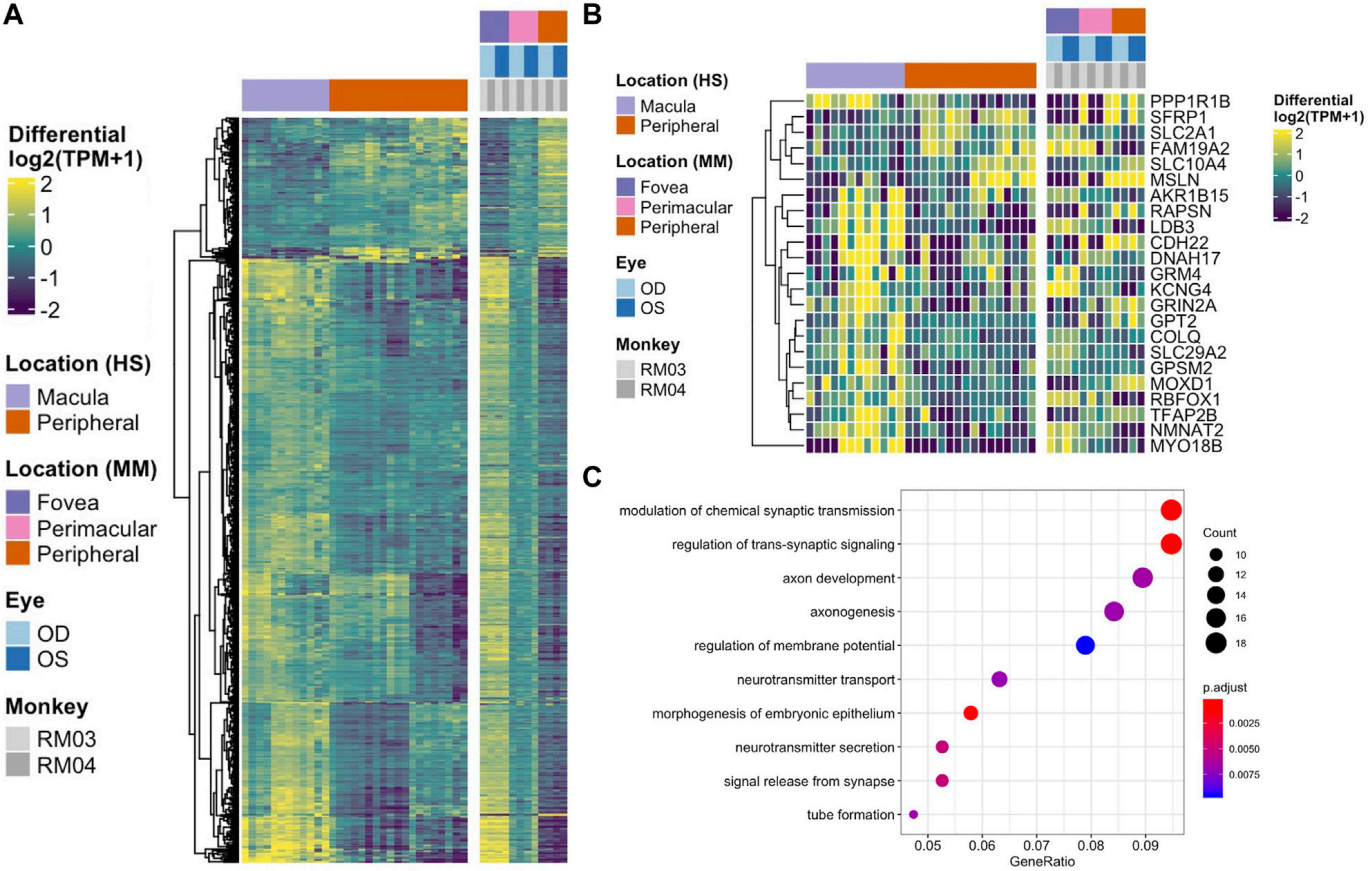
**(A)** Heatmap displaying the comparison of differential gene expression in the neural retina between published data from a human macula vs. periphery set (Li et al., 2014; Whitmore et al., 2014) and the macaque data presented here, with 664 genes following similar expression patterns. **(B)** A comparison of conserved differential expression to the human studies in the RPE/choroid tissue. Fewer genes follow similar patterns of differential expression. **(C)** GO enrichment for conserved differentially expressed genes between human and macaque datasets in the neural retina. Terms including neuronal connectivity and cellular morphogenesis are highly enriched.

To determine the conservation of location-specific differential expression of transcriptional regulators across fovea/macula to periphery, we performed a meta-analysis comparing our macaque data to previously published human datasets (Li et al., 2014; Whitmore et al., 2014). We see generally conserved patterns of expression from fovea/macula to periphery in neural retina and to a lesser extent, RPE/choroid (Figures 3A,B). We performed the same analysis on unfiltered gene lists from the human and macaque and found approximately 700 genes that follow similar differential expression patterns from fovea/macula to periphery in both species. (Figure 3A; Supplementary Table S1). GO terms for this list of conserved genes involve neuronal connectivity and cellular morphogenesis (Figure 3C), similar to the GO terms for the macaque retina data seen previously in this study. In one case, we see a distinct block of macula-specific transcription factors in the Rhesus monkey neural retina including *HR*, *MYC*, *EBF3*, *AR*, *EBF2*, *EBF1*, *TWIST1*, *IRX2*, *POU4F2*, *IRX1*, and *SHOX2* that seem to also correlate to macula-specific transcription factors in the human data (Supplementary Figure S6B). Overall, this supports a conservation of gene expression and function across monkey and human species.

**FIGURE 4:**
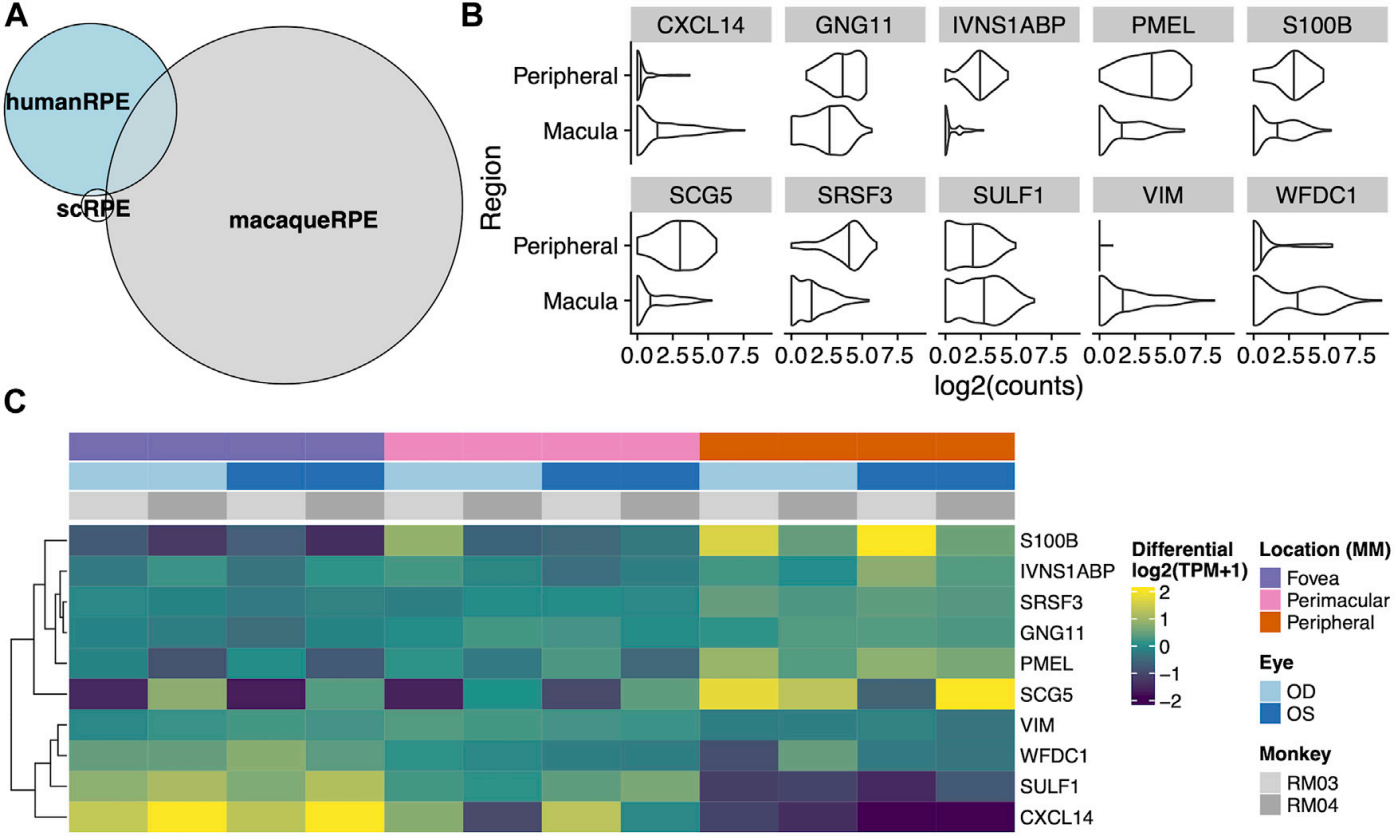
**(A)** Venn diagram of overlaps between differentially expressed genes between the fovea and periphery in three systems: Human single cell RPE, bulk human RPE-choroid, and bulk macaque RPE-choroid. **(B)** Violin plot of the single cell expression with the ten genes which overlap between the human single cell RPE testing and bulk macaque RPE-choroid testing. **(C)** Heatmap of the same ten genes in our macaque RPE-choroid punches.

To test whether there was any correspondence between our macaque tests and an independent single cell RPE cells taken from either the fovea/macula or periphery, we extracted 219 cells from the scEiaD resource at plae.nei.nih.gov. We found ten genes that overlap between our macaque resource and the scEiaD resource: *CXCL14, GNG11, IVNS1ABP, PMEL, S100B, SCG5, SRSF3, SULF1, VIM,* and *WFDC1*. Of these ten overlapping genes, *CXCL4*, *SULF4*, and *WFDC1* are shown to be enriched in the macula, and S100B, PMEL, and SCG5 are enriched in the periphery (Figure 4).

## Discussion

Using the Rhesus macaque as a model to study the transcriptome landscape of higher primate retina, we conducted RNA-sequencing across neural retina and RPE/choroid tissue layers. The small RNA punch size and bulk processing of the tissues allowed for high resolution and sensitivity of transcript quantification due to the decreased likelihood of variability from extended amplification, gene dropouts, and biological noise. Furthermore, it allows for the ability to profile rare and low-expressed transcripts as compared to single-cell RNAseq such as the 10x Genomics Chromium platform, in which only the top 20%–30% of expressed genes are captured, or Drop-seq, which requires a more manual approach. Unlike previous studies, our approach included the perimacular retina to define contiguously changing gene expression across the retinal landscape, thereby reflecting the stepwise differences in the ratio of cone and rod composition. By doing this, we isolated the critical pathways regulating changes in retinal connectivity and RPE/choroid function necessary to support different photoreceptor mosaics.

The macaque gene expression changes observed in our data are orthogonally confirmed in human datasets. Furthermore, our findings also indicate conservation of contiguously changing gene expression patterns from the central to peripheral retina between Rhesus monkeys and humans, establishing a more available model system for future human transcriptomic studies. Rhesus monkeys offer an accessible model in which conditions of tissue acquisition can be further controlled, including age, optimization of biopsy and RNA extraction, and reduced time from enucleation to tissue dissection and RNA extraction.

PCA of gene expression data by tissue type predicts the distinct nature of the neural retina and RPE/choroid tissues, and further clustering by location confirms there are transcriptomic differences between regions of the retina. The grouping of transcriptomes by animal highlights the genetic variability observed between individuals. The lower number of differentially expressed genes in fovea/macula vs. perimacular comparisons versus fovea/macula vs. periphery indicates a continuum of gene expression. The highly variable patterns of gene expression across the neural retina corresponds to the changing composition of cell types based on location.

In contrast, the presence of distinct changing RPE/choroid gene expression suggests variability in the cell processes occurring across the retina in the RPE and choroid. Due to the relatively unchanged morphology of these tissues across the retina save for increased RPE diameter and choroidal thickness in the macula, the contiguous changes in gene expression observed may be a factor in driving changes in cell processes across the entire retina. GO terms enriched for the contiguously changing genes in the retina involved neuron projection and axon development as well as regulation of cell morphogenesis, supporting known findings that there are differences in circuitry and cone packing across the neural retina (Jones et al., 2018). Furthermore, the changing gene expression across the RPE/choroid is enriched for GO terms such as cell junction organization and epithelial cell development, supporting reported findings that RPE diameter is smaller in the fovea/macula as compared to the periphery (R Sparrrow et al., 2010). It is known that RPE cells form tight junctions, creating the outer blood-retinal barrier. These tight junctions serve as regulators of cell proliferation, polarity, and transport, as well as transducers of signals responsible for regulating cell size and shape (Zou et al., 2020). Therefore, genes involved in cell junction organization and epithelial cell development may play a role in varied cell sizes across the RPE. GO terms involving urogenital and renal system development are also enriched in the RPE/choroid gene list, and interestingly, there are several heritable genetic conditions that affect both the eye and kidney, and there is also a link between chorioretinal thinning and chronic kidney disease, thought to be due to inflammation and endothelial dysfunction (Balmforth et al., 2016; Paterson et al., 2020). In addition to observing greater levels of gene expression variability across location in the RPE-choroid than expected, several disease-relevant genes known to play roles in various ocular disorders including *TIMP3* [various retinopathies (Dewing et al., 2020)], *ABCA4* [Stargardt disease (Walia and Fishman, 2009)], and *TYRP1* [oculocutaneous albinism (Simeonov et al., 2013)], were found to be significantly differentially expressed by location.

Differential expression of particular transcription factors, including neural retina- and RPE/choroid show conserved patterns of expression between macaque and human datasets, with *IRX2* being upregulated in the macula. Other differentially expressed transcription factors that show conservation with past human studies include *FOXI3*, which is upregulated in the periphery, and *POU4F2*, *POU4F1*, as well as its target, *RIT2*, all of which are enriched in the macula in both monkey and human datasets^7^. Additional transcription factors following similar gene expression patterns across species in the RPE/choroid include *VEZF1* and *NR2F1*, which are thought to be involved in retinal and vascular development (Tang et al., 2010; Zou et al., 2010). Identifying conserved location-specific transcription factors can provide insight into the regulatory landscape of the two tissues and how they relate to patterns of differential gene expression. It may be inferred then, that these changes in global gene expression across location and tissue, driven by distinct sets of transcription factors, likely reflect location-specific cellular processes and interactions.

Beyond patterns of transcriptional regulators, location-based tissue-tissue interactions are also indicative of specific pathways being up- or downregulated by location, suggesting specific roles in different locations of the tissues. The enrichment for Wnt signaling in kidney disease in the downregulated interactions in the macula may be connected to the GO enrichment for renal development in the RPE/choroid. Furthermore, ErbB signaling and peptide G-protein coupled receptor signaling are required for retinal development, with ErbB involved in neural crest development and adult pigment formation (Budi et al., 2008) and GCPR signaling involved in light processing in photoreceptors (Martemyanov, 2014). The tissue-tissue interaction analysis suggests these pathways are upregulated in the peripheral tissues of the eye. Certain pathways were enriched for tissue-tissue interactions both up- and downregulated in the macula, including Hippo signaling. Hippo-YAP signaling is known to play roles in both ocular development as well as disease, as it regulates retinogenesis, photoreceptor cell differentiation, and retinal vascular development, but misregulation also has associations with coloboma and optic fissure closure, uveal melanoma, and retinal degeneration (Lee et al., 2018). The downregulation of chemokine receptors observed in the fovea/macula may suggest a higher sensitivity to chemokine signaling in this region during disease, as AMD is known to be associated with upregulated levels of chemokine signaling (Newman et al., 2012). The widespread enrichment of this pathway informs the many roles it plays in regulating the retinal landscape across location.

The three-way comparison of single cell, bulk human, and Rhesus macaque data showed many more differentially expressed genes detected in our macaque dataset than the other two sequencing methods. It remains unclear whether this subset of differentially expressed genes without parent conservation in our dataset is identifying a Rhesus macaque-specific set of genes or if some of these genes are novel identifications due to the higher resolution of sequencing achieved in this dataset. Further investigation including functional studies are necessary to determine this. Furthermore, identifying differentially expressed genes in the Rhesus monkey data that intersect with the single cell data allows us to identify known RPE-specific genes that are conserved between the datasets including *VIM* (Zou et al., 2020), and *PMEL* (Reyes et al., 2020), which we have shown to change contiguously by location. Of the three macula-enriched genes in the overlapping set, *CXCL14* and *WFDC1* have been previously identified as enriched in the macula compared to peripheral retina *via* RNA microarray (Radeke et al., 2007), and *WFDC1* enrichment in the macula was independently confirmed by both expression and immunostaining (van Soest et al., 2007). As such, our data shows the known macula-enrichment of *WFDC1* to be conserved. As a serine protease inhibitor, mediator of endothelial cell migration and promoter of angiogenesis, its enrichment in the RPE and specifically, the macula, may suggest a role for *WFDC1* in age-related macular degeneration (Zhu et al., 2021), in which neovascularization beneath the macula is characteristic (Radeke et al., 2007). Additionally, *S100B,* a gene encoding a calcium-binding protein which we found enriched in the peripheral RPE has been linked with glaucoma, which can result in loss of peripheral vision (Kuehn et al., 2018).

Considering the differential gene expression patterns across contiguous regions of the neural retina and especially the RPE/choroid allows for a combined approach in which we assessed the drivers of change as well as gross changes in gene expression by location. We identified highly enriched gene ontologies associated with each tissue location and layer, highlighted sets of contiguously changing transcription factors, determined important tissue-tissue interactions that highlight various up- and downregulated location-specific pathways, and examined the conservation of gene expression patterns across multiple independent studies. In addition to the previous RNA-sequencing studies performed on human and non-human primate retinas, the data and findings presented here provide valuable resources for future studies aimed at identifying regional specialization of the retina and understanding disease mechanisms.

## Data Availability

The datasets presented in this study can be found in online repositories. The names of the repository/repositories and accession number(s) can be found in the article/Supplementary material.
