# Supplementary material for: Transcriptional mapping of the macaque retina and RPE-choroid reveals conserved inter-tissue transcription drivers and signaling pathways
Source: Front Genet. 2022 Nov 25;13:949449. doi: 10.3389/fgene.2022.949449 (PMC9732541; doi:10.3389/fgene.2022.949449)
Supplement: Supplementary file 1 [file DataSheet1.docx]

**Figure S1. (A)** Layout of punch biopsies taken from the Rhesus monkey eyes, including foveal (F), perimacular (PM), and peripheral (P) samples. **(B)** Diagram of the retina, highlighting the cell types in the neural retina as well as the RPE and choroid tissues (Adapted from “Structure of the Retina”, by BioRender.com (2022). Retrieved from <https://app.biorender.com/biorender-templates>).


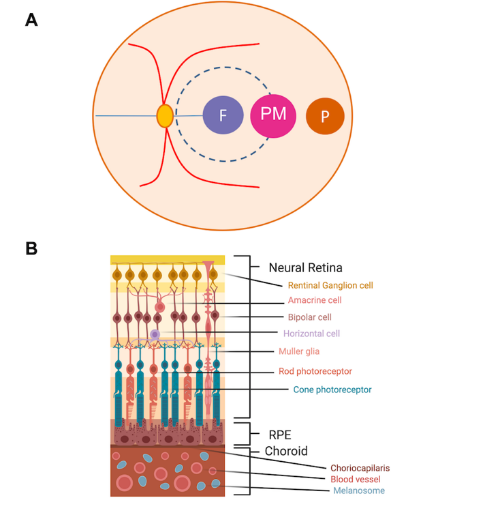


**Figure S2.** PCA clustering for the 1000 most variable genes sequenced. **(A)** Samples primarily cluster by tissue type, with RPE/choroid samples clustering away from retina samples. **(B)** Within the retina and RPE/choroid, foveal and peripheral samples tend to cluster furthest apart with perimacular samples clustering less tightly between.


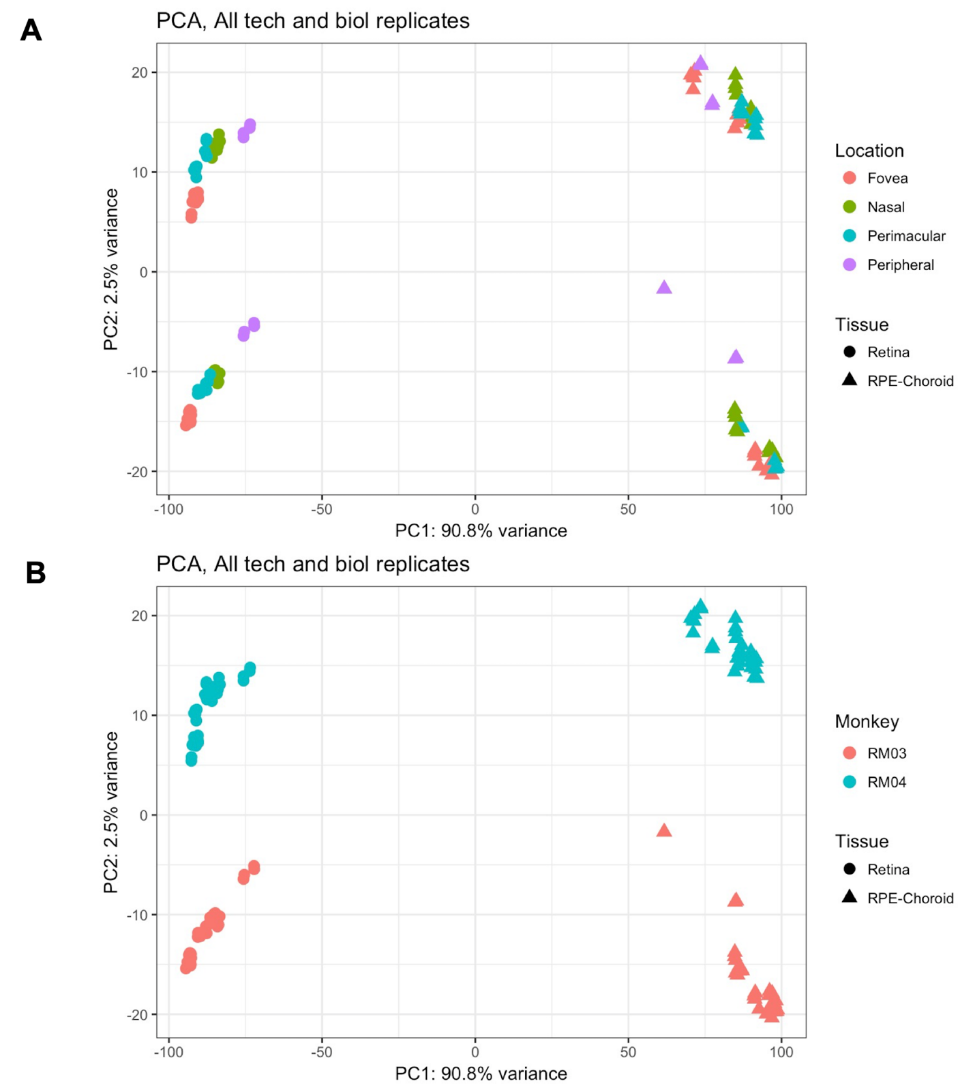


**Figure S3. (A)** Heatmap depicting macaque expression of published rod-enriched gene expression (Holt et. al 2015, Mustafi et. al, 2016) across the neural retina reflects cellular composition of rods in the retina. Genes are highly expressed in the periphery, where rod composition is highly enriched, and lowly expressed in the fovea/macula which is highly cone-enriched. **(B)** Peripheral and perimacular retinal samples express rod-enriched genes at a 2.8- and 1.7-fold increase compared to foveal retinal samples. The periphery is 1.6-fold enriched in cone signal compared to the fovea in the neural retina (Table S1). **(C)** Changes in rod- and cone-enriched gene expression do not vary greatly by location in RPE/choroid.


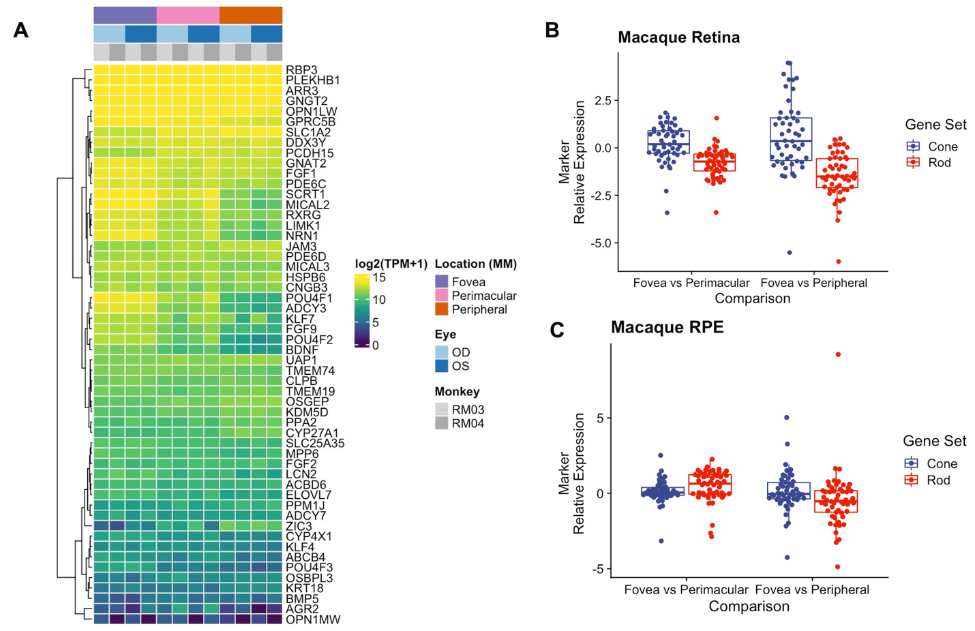


**Figure S4. (A)** Heatmap showing contiguous differential expression across location of the neural retina tissue, also exhibiting distinct blocks of progressive up and down regulated gene expression as seen for the RPE/choroid in Fig. 1C. **(B)** A subset of genes identified as differentially expressed transcription factors by location in the neural retina. Changes in transcriptional regulators can be a factor in driving gene expression changes.

*These transcription factors have published associations with the respective tissue in the literature. **(C)** Top 10 enriched gene ontology (GO) terms from the retina differentially expressed gene set, including neuron projection, axon development, regulation of cell morphogenesis, and protein localization and transport.


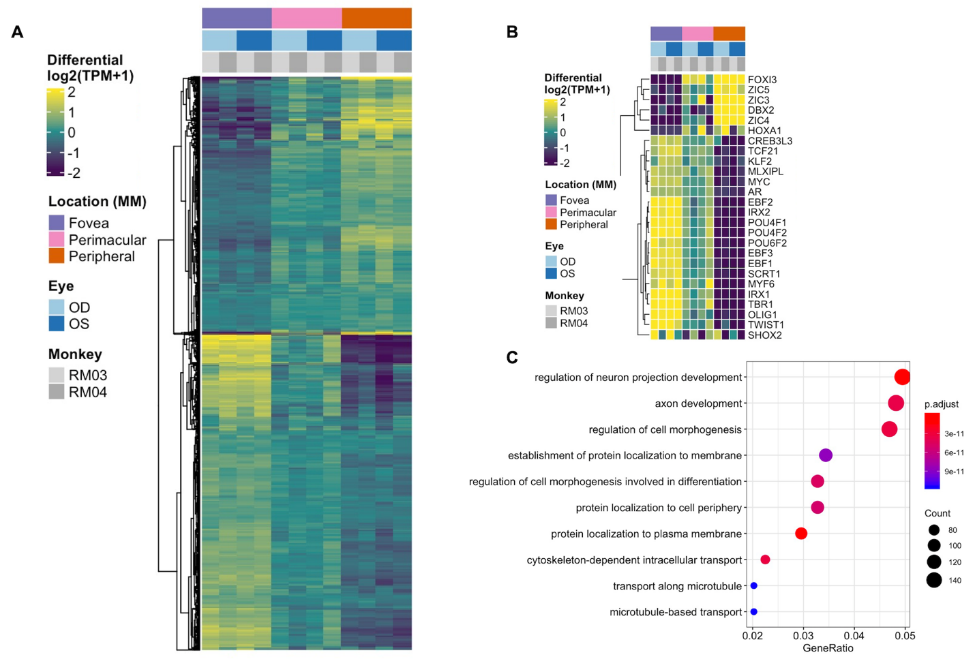


**Figure S5. (A)** Expression of commonly expressed transcription factors between the neural retina and the RPE/choroid. While *NR2F1* and *IRX2* follow similar expression patterns across location, *ZFHX4* actually displays opposite expression patterns in the retina and RPE/choroid tissues. **(B)** Expression of these three transcription factors from the publicly available EyeIntegration database (Swami and McGaughey et al., 2019) showing similar expression levels of each gene in both tissue types with whole blood for comparison.


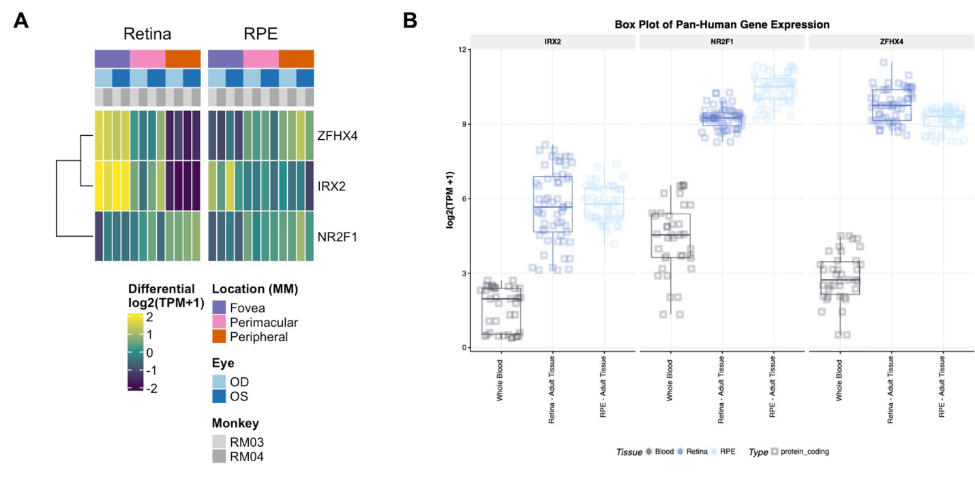


**Figure S6. (A)** A comparison of conserved transcription factor differential expression in the neural retina between published data from a human macula vs periphery set (Li et al., 2014, Whitmore et. al, 2014) and the macaque data presented here. **(B)** A similar comparison of human conservation of transcription factor differential expression in the RPE/choroid tissue. **(C)** Top enriched GO terms for the conserved gene expression across fovea to periphery in the RPE/choroid. Top terms include regulation of neurotransmitter levels and metabolic processes as well insulin secretion.


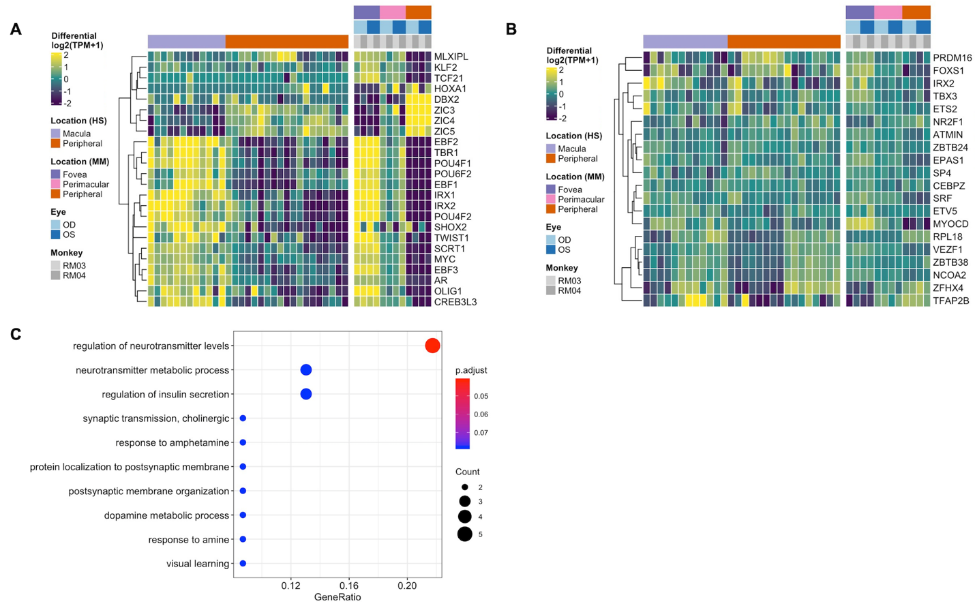


**Figure S7. (A)** Enrichr[1-3] bar graph depicting highly enriched pathways via the WikiPathways 2019 Human database for the downregulated ligand/receptor interactions in the macula. Top enriched pathways include Wnt signaling in kidney disease and ErbB signaling. **(B)** Enrichr clustergram depicts the genes contributing to the enriched pathway terms including *WNT*, *FZD4*, and *ERBB3*. **(C)** Enrichr bar graph for upregulated ligand/receptor interactions in the macula. Top enriched pathways include PI3K-Akt signaling and Focal Adhesion-PI3K-Akt-mTOR, both of which are involved in the VEGF signaling pathway. **(D)** Enrichr clustergram is enriched for genes including *VEGFA*, *FLT1*, and *KDR*, all of which are key components of VEGF signaling.


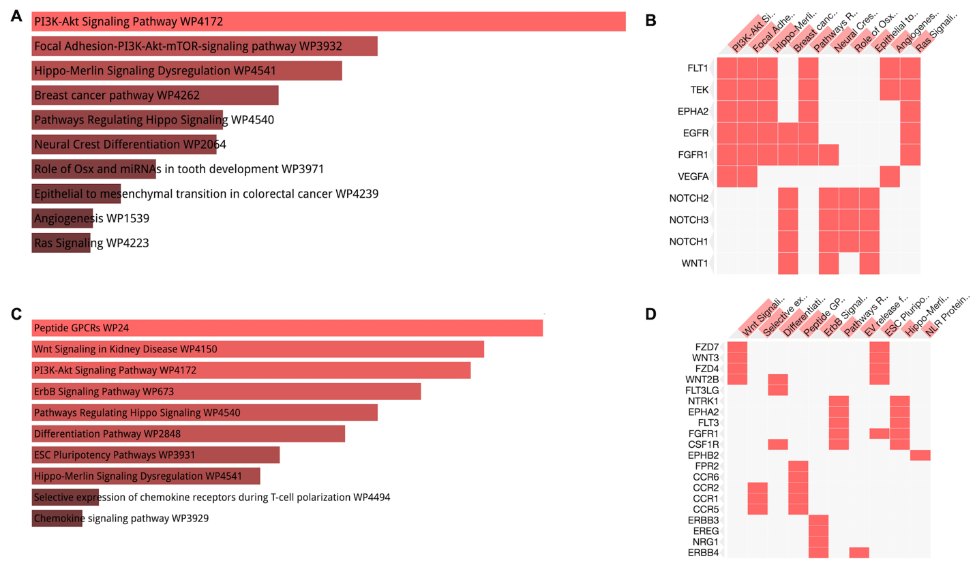


1. Chen, E.Y., et al., *Enrichr: interactive and collaborative HTML5 gene list enrichment analysis tool.* BMC bioinformatics, 2013. **14**(1): p. 1-14.

2. Kuleshov, M.V., et al., *Enrichr: a comprehensive gene set enrichment analysis web server 2016 update.* Nucleic acids research, 2016. **44**(W1): p. W90-W97.

3. Xie, Z., et al., *Gene set knowledge discovery with enrichr.* Current protocols, 2021. **1**(3): p. e90.
